# Draft Genome Sequence of Plant Growth-Promoting *Streptomyces* sp. Strain SA51, Isolated from Olive Trees

**DOI:** 10.1128/MRA.00768-19

**Published:** 2020-01-02

**Authors:** Sai Shiva Krishna Prasad Vurukonda, Mauro Mandrioli, Greta D’Apice, Emilio Stefani

**Affiliations:** a Department of Life Sciences, University of Modena and Reggio Emilia, Reggio Emilia, Italy; b Department of Life Sciences, University of Modena and Reggio Emilia, Modena, Italy; University of Arizona

## Abstract

A streptomycete was isolated from the rhizosphere of olive trees in the autumn of 2004. Its molecular characterization showed the presence of metabolic pathways promoting plant growth and additional properties that indicate that this strain is a prospective agent for future biocontrol applications *in planta*. We report here the draft genome sequence of Streptomyces avermitilis strain SA51.

## ANNOUNCEMENT

Plants are extensively colonized by a range of microorganisms, and such plant-microbe interactions may affect plant fitness and productivity. Indeed, the roots of many plants are infected and colonized by specific fungi (mycorrhizal association), rhizobia, and actinobacteria that help the plant acquire nutrients from the soil (1–3). Actinobacteria, and streptomycetes in particular, are mostly important in plant (root) interactions with other soil components. *Streptomyces* spp. influence soil fertility through the involvement of many biotic and abiotic components and serve as nutrient uptake and plant growth enhancers. Streptomycetes are known to solubilize phosphates and produce siderophores; additionally, they synthesize and export enzymes like amylase, chitinase, cellulase, invertase, lipase, keratinase, peroxidase, pectinase, protease, phytase, and xylanase, which change the complex soil nutrients into simple mineral forms. This nutrient cycling capacity makes them ideal candidates for natural biofertilizers (4–7).

In the present study, the draft genome of strain SA51, isolated from the rhizosphere of an olive tree, has been characterized. Rhizospheric soil samples were collected and suspended in a sterile saline solution; suspensions were serially diluted, and replicates of 50-μl samples were plated on International Streptomyces Project medium 2 (ISP-2) agar and incubated for 7 days at 28°C (8). Colonies resembling those of streptomycetes were purified on the same medium and checked for antagonistic activity against a set of phytopathogenic bacteria and fungi, and their plant growth promotion properties were evaluated on tomato as a model plant. Strain SA51, as the most active streptomycete, was subcultured three times on ISP-2 agar prior to DNA extraction. For genomic DNA extraction, single colonies of Streptomyces sp. strain SA51 were grown in tryptic soy broth (TSB) for 3 days at 28°C. Genomic DNA was extracted and purified using the DNeasy blood and tissue kit (Qiagen, Hilden, Germany), and its quantity and quality were checked using the NanoDrop One microvolume UV-visible (UV-Vis) spectrophotometer (Thermo Fisher Scientific, Waltham, MA, USA), followed by gel electrophoresis. DNA sequencing was performed using an Illumina HiSeq 2000 sequencer. High-quality Illumina sequence libraries were prepared using the Nextera DNA Flex library prep kit. Genome assembly from paired-end sequence reads was done using the default parameters of the assembler module available in the Geneious software v1.0 that includes quality control, trimming, and assembly steps, using default parameters. Sequence alignment was done using the ClustalW and “Map to a reference” tools available in Geneious v1.0. Coverage was determined by alignment to the *Streptomyces avermitilis* reference genome (GenBank accession number NC_003155) to be 30×, whereas the coverage breadth was 95.3%. The total genome size of strain SA51 is 5,465,072 bp (including 792 assembled contigs and 2,959 unassembled reads), with a GC content of 70.1%. The mean contig length was 2,832 bp, whereas the shorter and longer contigs were 707 bp and 23,079 bp, respectively (*N*_50_ length, 3,517 bp) (Table 1).

**TABLE 1:** Strain SA51 genome contig assembly report

Statistic	Value for:			
	Unassembled reads	All contigs	Contigs ≥100 bp	Contigs ≤1,000 bp
Total no.	2,959	792	792	74
Minimum length (bp)	398	707	707	1,001
Median length (bp)		2,179	2,325	2,179
Mean length (bp)	1,088	2,832	2,832	2,969
Maximum length (bp)	14,732	23,079	23,079	23,079
*N*_50_ length (bp)		3,517	3,517	3,565
No. of contigs ≥ *N*_50_ value		199	199	193
Length sum (bp)	3,221,808	2,243,264	2,243,264	2,197,473

Annotation and subsystem coverage analysis were performed using the Rapid Annotation using Subsystems Technology (RAST) server, with standard parameters (9) provided by the SEED project (10). Genome annotation with RAST identified 6,040 coding sequences (CDSs), 32 tRNAs, and 13 rRNAs in the SA51 genome. Amplification of the short subunit (SSU) 16S rRNA was carried out using the primer pair strepB (5′-ACAAGCCCTGGAAACGGGGT-3′) and strepE (5′-CACCAGGAATTCCGATCT-3′) (11). The amplification was carried out in a 25-μl total volume. PCRs were performed with 1× GoTaq Buffer, 0.8 μM each of forward and reverse primer, 2 μl DNA template, 200 μM deoxynucleoside triphosphates (dNTPs), 1.250 mM MgCl_2_, and 1 U Taq polymerase; the remaining volume was added with nuclease-free water. PCR conditions started with predenaturation (94°C, 5 min), followed by denaturation (94°C, 60 s), annealing (55°C, 60 s), elongation (72°C, 1 min 30 s), and postelongation (72°C, 5 min). Sanger sequencing of the 16S rRNA gene PCR product, followed by nucleic acid sequence BLAST analysis (https://blast.ncbi.nlm.nih.gov/Blast.cgi) of the strains present in NCBI GenBank, revealed that strain SA51 was identified as Streptomyces avermitilis, with an identity of 97% and an E value of 0.3E−77.

In order to identify genes involved in plant growth promotion, we constructed the SA51 metabolic profile using the Kyoto Encyclopedia of Genes and Genomes (KEGG) (12), thus providing evidence for the presence of genes involved in the pathway for indole alkaloid biosynthesis and in iron transport and metabolism, together with genes coding for proteins acting in the regulation of iron homeostasis. At the same time, based on RAST annotations, we provided evidence for the presence of genes and operons related to metal transporters and antibiotic biosynthesis, suggesting that SA51 could be involved in the biological control of plant pathogens and/or in the reshaping of the soil microbiota.

Overall, these preliminary studies suggest that S. avermitilis strain SA51 deserves additional study and provide insight into its capability to act as a growth-promoting microorganism in agricultural systems, together with its possible role in supporting the plant resistome.

### Data availability.

The draft genome sequence was deposited at DDBJ/ENA/GenBank under BioProject number PRJNA545025 and accession number VEXM00000000. The version described in this paper is the first version, VEXM01000000. The fastq files of the raw reads were deposited in the NCBI Sequence Read Archive (SRA) under accession number SRR10416223.
